# Perinatal outcomes in anemic pregnant women in public hospitals of eastern Ethiopia

**DOI:** 10.1093/inthealth/ihac021

**Published:** 2022-04-26

**Authors:** Adera Debella, Addis Eyeberu, Tamirat Getachew, Genanaw Atnafe, Biftu Geda, Merga Dheresa

**Affiliations:** School of Nursing and Midwifery, College of Health and Medical Sciences, Haramaya University, Harar, Ethiopia; School of Nursing and Midwifery, College of Health and Medical Sciences, Haramaya University, Harar, Ethiopia; School of Nursing and Midwifery, College of Health and Medical Sciences, Haramaya University, Harar, Ethiopia; Department of Pediatrics, School of Nursing and Midwifery, College of Health and Medical Sciences, Haramaya University, Harar, Ethiopia; School of Nursing, College of Medicine and Health Sciences, Madda Walabu University, Bale-Robe, Ethiopia; School of Nursing and Midwifery, College of Health and Medical Sciences, Haramaya University, Harar, Ethiopia

**Keywords:** anemia, Ethiopia, newborn, perinatal outcome, pregnant

## Abstract

**Background:**

Anemia is a worldwide problem with serious effects for mothers and their babies. Although efforts have been made to lessen the burden of anemia, it has remained a problem. Moreover, there is a paucity of information regarding the perinatal outcomes of anemia in the study area. Thus this study aimed to assess the perinatal outcomes in anemic pregnant women in eastern Ethiopia.

**Methods:**

A facility-based cross-sectional study was conducted among 407 systematically selected pregnant women. Data were collected by interview and entered into EpiData version 3.1 and then exported into SPSS for Windows version 20 for analysis. Bivariate and multivariate analyses were employed to determine the association between independent variables and the outcome variable.

**Results:**

Among pregnant women, 61.9% had an adverse perinatal outcome. The most common reported adverse perinatal outcomes were preterm birth, congenital anomalies and stillbirths. Furthermore, variables such as educational status (adjusted odds ratio [AOR] 2.11 [95% confidence interval {CI} 1.245 to 3.58]), antenatal care follow-up (AOR 2.75 [95% CI 1.47 to 5.18]) and hemoglobin level (AOR 4.1 [95% CI 2.609 to 6.405]) were significantly associated with perinatal outcomes.

**Conclusions:**

Nearly three-fourths of anemic pregnant women experienced adverse perinatal outcomes. In general, this study identified that educational status, antenatal follow-up and hemoglobin level were associated with perinatal outcomes among anemic pregnant women. To prevent adverse perinatal outcomes, efforts must be made to ensure that all pregnant women receive antenatal care and have adequate maternal nutritional status.

## Introduction

Anemia is a condition in which the hemoglobin concentration in the blood is lower than normal.^1^ There are numerous causes of anemia, but dietary deficiencies are the most common. Anemia affects 40% of the world's population.^2,3^

Although anemia causes severe morbidity and mortality in all segments of the population, pregnant women are the most vulnerable population due to their unique physiological state. Globally, 38.2% of pregnant women are anemic.^4^ In Africa, 48% of reproductive-age women and 56% of pregnant women are affected by anemia.^5,6^ In Ethiopia, nearly one-third of pregnant women are anemic.^7^

Anemia is a global problem with serious consequences for mothers and their children. Evidence suggests that postpartum hemorrhage, postpartum febrile morbidity and sepsis are among the adverse perinatal outcomes observed in anemic pregnant women.^8^ Anemia during pregnancy increases the risk of preeclampsia, placenta previa, cesarean delivery and preterm labor.^9^ Similarly, maternal anemia increases the risk of maternal and perinatal deaths.^10^ Each year, 115 000 maternal deaths and 591 000 perinatal deaths are recorded.^6^

Maternal anemia also has short-term and far-reaching sequelae for the newborn. Anemia is associated with morbidity and mortality of growing fetuses and neonates.^11,12^ It leads to premature birth, low birthweight, fetal cognitive impairment and death.^13–15^ The perinatal adverse outcomes of anemia include low birthweight (12%), preterm births (19%) and perinatal mortality (18%).^16^

The World Health Organization has implemented a global nutritional strategy to reduce anemia by 50% by 2025 as an important component of the health of women and children, which in turn plays a pivotal role in combating the adverse outcomes of anemia.^1,3^ Similarly, the Federal Ministry of Health of Ethiopia has launched a national nutritional strategy to end iron deficiency anemia.^17^ Despite several international and national interventions, anemia remains a problem.

Moreover, there is a paucity of research regarding maternal anemia and its association with perinatal outcomes at the study area level to help those working on the problem propose evidence-based measures. Furthermore, studies have shown that the relationship between maternal anemia and perinatal outcomes is unclear.^18^ Some studies have shown a strong association between low hemoglobin before delivery and adverse outcome, while other studies have not found a significant association.^18–20^ Therefore this study was conducted to investigate the association between anemia and perinatal outcomes in anemic pregnant women in eastern Ethiopia.

## Methods

### Study area and period

The study was conducted in four public hospitals of Dire Dawa city administration and Harari regional state. The two public Hospitals namely Hiwot Fana Specialized University Hospital and Jugol General Hospital located in Harari Regional State, whereas two public hospitals such as Dilchora Referral Hospital and Sabian Primary Hospital found in the Dire Dawa city administration, eastern Ethiopia. Harari regional state is found at a distance of 526 km to east of the capital city. There are two military, two public and two private hospitals and eight health centers.^21^ While the Dire Dawa city administration is one of the federal city administrations found 515 km to the east of Addis Ababa. There are two government hospitals namely Dilchora Referral Hospital and Sabia Primary Hospital and 17 health centers that provide services for approximately 1.5 million people in the catchment area. The study had been conducted from 20 January to 19 February 2018.

### Study design and population

An institutional-based cross-sectional study was conducted among randomly selected mothers who came to give birth in public hospitals in Harar regional state and Dire Dawa city administration. Pregnant women with a history of preterm delivery, women with a sonographic diagnosis of congenital malformation, obstetrical complications such as pregnancy-induced hypertension and multiple pregnancies were excluded from the study.

### Sample size determination and sampling procedures

The sample size was calculated by using a single population proportion formula with assumptions of a 95% confidence level of 1.96, a margin of error (d) of 0.05 and a prevalence of prenatal anemia among pregnant women of 0.361 from a previous study conducted at antenatal clinics in the northwestern zone of Tigray^22^ and adding a 15% nonresponse rate. The final sample size was 407. A systematic random sampling method was used to select the study participants. A proportional allocation was employed to obtain the sample size from the public hospitals. The first sample was selected by the lottery method.

### Data collection methods

Data were collected using a pretested interviewer-administered questionnaire adopted from previous literature.^23–25^ It consists of sociodemographic (age, marital status, monthly income, religion, ethnicity, occupation), maternal and neonatal characteristics. The questionnaire was initially prepared in English and translated to the local languages (Afan Oromo, Amharic and Somali). It was then translated back to English by to check its consistency. The questionnaire was pretested on 5% of the sample size of the study area to ensure its validity. A blood sample was collected from each participant. Each woman was interviewed during the intrapartum period while she was in relatively stable condition. The data were collected by 12 well-trained bachelor of science–holding midwives. To diagnose anemia, the hemoglobin level of the mothers was measured using an HB 301 analyzer (HemoCue, Ängelholm, Sweden). A blood sample was collected through a finger prick by sterile lancet and dropped on a microcuvette for analysis.

### Statistical analysis

The data were coded, edited, cleaned and entered into EpiData statistical software version 3.1 (EpiData Software, Odense, Denmark) and then exported to SPSS version 20 (IBM, Armonk, NY, USA) for analysis. A descriptive statistical analysis was used to summarize the data. The information was presented using frequencies, tables and figures.

Bivariate analysis and multivariate analysis were done to see the association between each independent variable and outcome variables by using binary logistic regression. The assumptions of binary logistic regression were cheeked. The goodness of fit was checked by the Hosmer–Lemeshow statistic and omnibus tests. All variables with a p-value <0.25 in the bivariate analysis were included in the final model of the multivariate analysis to control all possible confounders. A multicolinearity test was carried out to see the correlation between independent variables by using the standard error and collinearity statistics (variance inflation factors >10 and standard error >2 were considered as suggestive of the existence of multicolinearity). The direction and strength statistical association was measured by the odds ratio (OR) with 95% confidence interval (CI). The adjusted odds ratio (AOR) along with the 95% CI were estimated to identify the association between independent variables and perinatal outcome by using multivariate analysis in binary logistic regression. In this study, p-values <0.05 were considered statistically significant.

### Ethical considerations

Ethical clearance was provided by the Institutional Health Research Ethics Review Committee of the College of Health and Medical Sciences of Haramaya University. In addition, permission to proceed was obtained from all public hospitals and informed written and signed consent was obtained from each participant.

## Results

### Sociodemographic characteristics

A total of 405 study participants were involved and yielded a response rate of 99.5%. The mean age of the study participants was 26.6±6.157. Most of the participants (321 [79.2%]) were married, 131 (32.3%) were unable to read and write and 248 (61.2%) were urban residents (Table 1).

**Table 1.** Sociodemographic characteristics of study participants in public hospitals of Harar regional state and Dire Dawa city administration, eastern Ethiopia, 2018

Variables	Categories	n	%
Age of participant (years)	15–24	145	35.8
	25–34	206	50.9
	35–44	54	13.3
Residence	Urban	248	61.2
	Rural	157	38.8
Marital status	Married	321	79.2
	Divorced	21	5.2
	Single	34	8.4
	Separated	29	7.2
Educational status	Unable to read and write	131	32.3
	Able to read and write	24	5.9
	Primary level	77	19
	Secondary level	88	21.7
	College or above	85	21
Occupational status	Housewife	199	49.2
	Government employee	61	15
	Private employee	52	12.8
	Daily laborer	19	4.7
	Merchant	42	10.3
	Student	32	8
Income (birr)	None	152	37.5
	<1000	46	11.4
	1000–2000	82	20.2
	≥2000	125	30.9

### Perinatal outcome

Among the study participants, 159 (39.3%) had an adverse perinatal outcome. From a total of 90 (22.2%) participants, 6 (1.5%) neonates were born with congenital anomalies, 20 (4.9%) mothers had a stillbirth and 81 (20%) and 40 (9.9%) newborns had an Apgar score <7 in at 1 and 5 min, respectively (Table 2).

**Table 2.** Perinatal outcome among the laboring mothers admitted to public hospitals of Harari regional state and Dire Dawa city administration, eastern Ethiopia, 2018

Variables	Categories	n	%
Gestational age (weeks)	Pre-term (<37)	90	22.2
	Term (≥37)	315	77.8
Birth outcome	Live birth	385	95.1
	Stillbirth	20	4.9
Sex of the baby	Male	219	54.1
	Female	186	45.9
Mode of delivery	Vaginal	298	73.6
	Caesarian section	80	19.8
	Instrumental	27	6.7
Apgar score (1 min)	<7	81	20
	≥7	324	80
Apgar score (5 min)	<7	40	9.9
	≥7	365	90.1
Birthweight (g)	Low birthweight (<2500)	76	18.8
	Normal birthweight (≥2500)	329	81.2
Congenital anomalies	Yes	6	1.5
	No	399	98.5
Adverse perinatal outcome	Yes	159	39.3
	No	246	60.7

**Figure 1.**
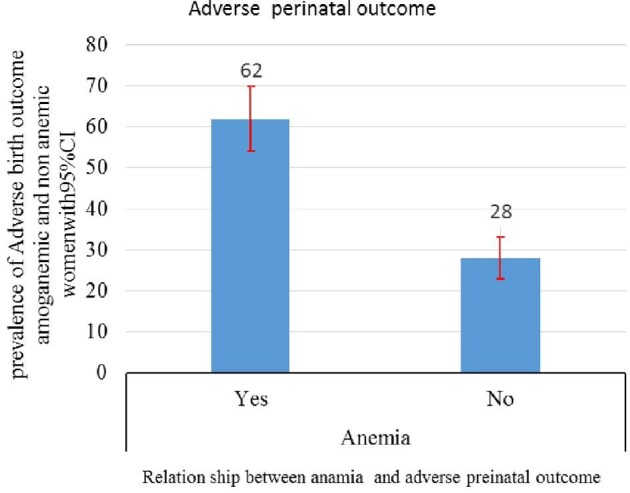
The relationship between anemia and adverse perinatal outcome among pregnant women admitted to Public Hospital of Harari regional state and Dire Dawa city administration, eastern Ethiopia, 2018.

A neonate born to an anemic women is more likely to have adverse perinatal outcomes as compared with those born to nonanemic pregnant mothers. Among 134 (33.1%) pregnant women who had anemia, 83 (61.9%) developed adverse perinatal outcomes. In contrast, among 171 (66.9%) women who did not have anemia, only 76 (28%) developed an adverse perinatal outcome (Figure 1).

### Maternal and obstetric characteristics

A total of 327 (80.7%) mothers had an antenatal care (ANC) follow-up and among those mothers who had ANC follow-up, 71.6% received iron and folic acid supplements (Table 3).

**Table 3.** Maternal and obstetric characteristics among pregnant women admitted to public hospitals of Harari regional state and Dire Dawa city administration, eastern Ethiopia, 2018

Variables	Categories	n	%
ANC follow-up	Yes	327	80.7
	No	78	19.3
Gravidity	1	135	33.3
	2–4	191	47.2
	≥5	79	19.5
Parity	1	160	39.5
	2–4	181	44.7
	≥5	64	15.8
Blood loss in current pregnancy	Yes	38	9.4
	No	367	90.6
Hemoglobin (mg/dl)	<11	134	33.1
	≥11	271	66.0
Iron and folic acid intake during current pregnancy	Yes	290	71.6
	No	115	28.4
Duration of iron and folic acid intake (months)	1–4	232	57.3
	≥4	58	14.3
Pregnancy-induced hypertension	Yes	11	2.7
	No	394	97.3

### Factors associated with perinatal outcomes

Variables such as residence, educational status, income level, ANC follow-up and hemoglobin level were associated with perinatal outcomes in the bivariate logistic regression, but residency and income level become insignificant in the multivariate logistic regression analysis. Educational status, ANC follow-up and hemoglobin level were associated with perinatal outcomes among pregnant women.

Multivariate analysis indicated that women unable to read and write were 2.11 times (AOR 2.11 [95% CI 1.245 to 3.58]) more likely to have adverse perinatal outcomes than women who finished a primary level education or above. The odds of adverse perinatal outcomes among pregnant mothers who did not have ANC follow-up were 2.75 times greater than for mothers having ANC follow-up (AOR 2.75 [95% CI 1.47 to 5.18]) (Table 4).

**Table 4.** Factors associated with adverse perinatal outcome among pregnant women admitted to public hospitals of Harari regional state and Dire Dawa city administration, eastern Ethiopia, 2018

		Perinatal outcome			
Variables	Categories	Adverse	Normal	OR (95% CI)	AOR (95% CI)
Residence	Rural	86	71	2.9 (1.92 to 4.4)*	1.33 (0.79 to 2.23)
	Urban	73	175	1	1
Educational status	Unable to read and write	71	60	2.56 (1.66 to 3.96)	2.11 (1.245 to 3.58)*
	Able to read and write	9	15	1.30 (0.55 to 3.10)	1.23 (0.48 to 3.16)
	Primary level education or above	79	171	1	1
Income (birr)	None	59	93	1	1
	1–1000	23	23	1.45 (0.88 to 2.44)	0.75 (0.42 to 1.35)
	1001–2000	39	43	2.29 (1.15 to 4.58)	1.16 (0.52 to 2.60)
	>2001	38	87	2.08 (1.17 to 3.70)	1.3 3(0.69 to 2.54)
ANC follow-up	No	56	22	5.54 (3.21 to 9.55)	2.75 (1.47 to 5.18)*
	Yes	103	224	1	
Hypertension	No	58	77	1	1
	Yes	63	128	0.53 (0.16 to 1.76)	2.11 (0.55 to 8.06)
Gravidity	1	70	89	1	1
	2–4	58	123	0.600 (0.385 to 0.933)	1.35 (0.69 to 2.64)
	≥5	31	33	1.194 (0.668 to 2.137)	1.05(0.56 to 1.96)
Hemoglobin (mg/dl)	<11	83	51	4.176 (2.695 to 6.471)	2.92 (1.76 to 4.83)*
	≥11	76	195	1	

*p≤0.000.

Pregnant women with a hemoglobin level <11 g/dl were 4.1 times more likely to have adverse perinatal outcomes as compared with women with hemoglobin levels >11 g/dl (AOR 4.1 [95% CI 2.609 to 6.405]) (Table 4).

## Discussion

This study assessed the perinatal outcome in anemic pregnant women admitted in the public hospitals of Harari regional state and the Dire Dawa city administration. Additionally, factors associated with perinatal outcomes were identified.

The present study reported that preterm (22.2%), stillbirths (4.9%) and congenital anomalies (1.5%) were the common adverse fetal and neonatal outcomes in anemic pregnant women. Similarly, findings from other studies showed that preterm birth, low birthweight (<2500 g) and neonatal complications were identified as the common adverse perinatal outcomes in anemic pregnant women.^26,27^ Additionally, similar studies conducted in India, Pakistan and Tanzania showed that the adverse perinatal outcomes in anemic pregnant women were stillbirth, low birthweight, small for gestational age, early neonatal mortality and neonatal morbidity.^28–30^ The findings revealed that there is a clear gap in the prevention and management of anemia during pregnancy, despite the fact that these adverse outcomes are preventable and treatable conditions.^2^ Evidence suggests that effective contraception and routine antenatal iron supplementation in pregnancy are recommended preventive measures for the adverse perinatal outcomes of anemia.^31,32^ Similar evidence also shows the importance of improving maternal nutritional status and iron supplementation during pregnancy to reduce this adverse perinatal outcome.^33^

In this study, 20% and 9.9% of the newborns had an Apgar score <7 in at 1 and 5 min, respectively. This is in line with a study done in Pakistan.^34^ The possible justification is that newborns with a low Apgar score are more likely to develop neonatal complications, increasing the risk of perinatal morbidity and mortality.^35^ The study pointed out that a low Apgar score was found to be associated with increased mortality in premature neonates.^36^

In this study, respondents who were unable to read and write were 2.11 times (AOR 2.11 [95% CI 1.245 to 3.58]) more likely to have adverse perinatal outcomes than respondents who finished primary level education or above. This finding was supported by research done in low- and mid-income countries.^37,38^ One possible explanation is that those with a high level of education understand the importance of ANC follow-up and visit health facilities to seek health services. As a result, the adverse outcomes of the perinatal period will be reduced.

In this study, the odds of having adverse perinatal outcomes among pregnant mothers who did not have an ANC follow-up were 2.75 times those of mothers having an ANC follow-up (AOR 2.75 [95% CI 1.47 to 5.18]). In fact, those who had ANC follow-up received various services such as iron and folic acid supplementation, nutrition counseling and early detection and treatment of health problems,^39^ all of which are important in reducing adverse perinatal outcomes. According to the evidence, ANC follow-up allows women to receive appropriate prevention and treatment. Women who received ANC and iron supplementation had a lower risk of adverse perinatal outcomes.^26,32^

In this study, women who had a hemoglobin level <11 mg/dl were 4.1 times more likely to experience adverse perinatal outcomes as compared with those who had a hemoglobin level >11 mg/dl. This result is consistent with the study conducted in Hosanna town, in southern Ethiopia.^40^ These links may be explained by the fact that anemia has an effect on oxygen carrying capacity and its transportation to placental sites for the fetus. This suggested that pregnant women with anemia are more likely to have a poor perinatal outcome as compared with nonanemic women. Hence there were a greater number of cases among anemic women than among nonanemic women.

## Limitations of the study

The study could not establish a cause–effect relationship, as the study design was cross-sectional. A number of the factors were liable to social desirability bias. Taking the information while the women were in labor was comparatively difficult.

## Conclusions

Nearly three-quarters of pregnant women with anemia had adverse perinatal outcomes. In general, this study identified that educational status, ANC follow-up and hemoglobin level were associated with perinatal outcomes among pregnant women. Efforts are needed to ensure all pregnant women have ANC follow-up and adequate maternal nutritional status to prevent adverse perinatal outcomes. Similarly, control of maternal anemia and improving iron supplementation are important strategies to prevent adverse perinatal outcomes.

## Data Availability

All relevant data are included in this study. However, additional data are available from the corresponding author upon reasonable request.
